# Supplementary material for: Meningeal contrast enhancement in multiple sclerosis: Assessment of field strength, acquisition delay, and clinical relevance
Source: PLoS One. 2024 May 29;19(5):e0300298. doi: 10.1371/journal.pone.0300298 (PMC11135724; doi:10.1371/journal.pone.0300298)
Supplement: S1 Appendix — (DOCX) [file pone.0300298.s001.docx]

**S1 appendix**

*MRI Sequence Parameters*

7T FLAIR: TR = 8,000 ms, TE = 300 ms, TI = 2,200 ms, flip angle = 70-degrees, resolution = 0.500 x 0.488 x 0.488 mm3, SENSE acceleration factor = 2.5 x 3.0, and acquisition time = 9 minutes 4 seconds.

7T MP2RAGE: MP2RAGE TR = 8,250 ms, TR = 6.9 ms, TE= 1.97 ms, inversion times = 1,000/3,300 ms, flip angles = 5/5 degrees, Turbo factor = 252, resolution = 0.700 x 0.688 x 0.688 mm3, SENSE acceleration factor = 2 x 2, and acquisition time = 9 minutes 36 seconds.

3T FLAIR: TR = 5000 ms, TE = 390 ms, TI = 1800 ms, resolution = 0.5 x 0.5 x 1.0 mm3, GRAPPA = 2, acquisition time = 7 minutes, 27 seconds.

3T MP2RAGE: TR = 5000 ms, TE = 2.98 ms, Inversion times = 700/2500 ms, flip angles = 4/5 degrees, resolution = 1.0 x 1.0 x 1.0 mm3, GRAPPA = 3, acquisition time = 8 minutes 22 seconds.
