# Supplementary material for: Meningeal contrast enhancement in multiple sclerosis: Assessment of field strength, acquisition delay, and clinical relevance
Source: PLoS One. 2024 May 29;19(5):e0300298. doi: 10.1371/journal.pone.0300298 (PMC11135724; doi:10.1371/journal.pone.0300298)
Supplement: S1 Table — (DOCX) [file pone.0300298.s002.docx]

**S1 Table: Meningeal enhancement subtypes and total enhancement.**

|  | | MS | | | HC | | |
| --- | --- | --- | --- | --- | --- | --- | --- |
|  |  | Volume (mm^3^) | Count | ME Present (%) | Volume  (mm^3^) | Count | ME Present (%) |
| Nodular | Gd+ Delayed 3T FLAIR | 2.2 (11.3)  0^b^ [0-86] | 0.1 (0.4)  0^b,c^ [0-2] | 6^b,c^ (10) | 1.6 (5.4)  0 [0-22] | 0.1 (0.3)  0 [0-1] | 2 (12.5) |
|  | Gd+ Early 7T FLAIR | 5.73 (12.4)  0^b^ [0-76.9] | 0.4 (0.7)  0^b^ [0-3] | 31^b^ (32.6) | 2.1 (7.2)  0 [0-29.5] | 0.1 (0.3)  0 [0-1] | 2 (11.8) |
|  | Gd+ Delayed 7T FLAIR | 3.3 (7.9)  0 [0-45.8] | 0.5 (1)  0^c^ [0-5] | 28^c^ (29.5) | 3.1 (7.7)  0 [0-29.9] | 0.4 (1)  0 [0-4] | 4 (23.5) |
| Spread / Fill | Gd+ Delayed 3T FLAIR | 7.5 (18.6)  0^c^ [0-95] | 0.3^c^ (0.7)  0 [0-3] | 14^c^ (23.3) | 23.9 (60.2)  0 [0-218] | 0.4 (0.6)  0 [0-2] | 5 (31.3) |
|  | Gd+ Early 7T FLAIR | 119.3 (888.6)  0^d^ [0-8623] | 0.5 (1.2)  0^d^ [0-9] | 26^d^ (27.4) | 26.3 (50.2)  0  [0-137.9] | 0.4 (0.7)  0 [0-2] | 4 (23.5) |
|  | Gd+ Delayed 7T FLAIR | 69.9 (390.5)  0^c,d^ [0-3780.7] | 0.9 (1.6)  0^c,d^ [0-10] | 42^c,d^ (44.2) | 40.8 (106.9)  0 [0-420.2] | 0.6 (1.1)  0 [0-4] | 5 (29.4) |
| Paravascular | Gd+ Delayed 3T FLAIR | 419.9 (98414)  38.4^b,c^ [0-6505] | 2.2 (3.6)  1^b,c^ [0-19] | 34^b,c^ (56.7) | 323.1 (725.5)  0^c^ [0-2837] | 1.8 (2.9)  0^c^ [0-10] | 7 (43.8) |
|  | Gd+ Early 7T FLAIR | 643.2 (999.3)  203.4^b,d^ [0-4955.1] | 3.4 (3.7)  2^a,b,d^ [0-18] | 72^b,d^ (75.8) | 143.9 (213.4)  40.7^a^ [0-728.5] | 1.3 (1.7)  1^a,d^ [0-6] | 9 (52.9) |
|  | Gd+ Delayed 7T FLAIR | 1185.7 (2058.9)  599.2^c,d^ [0-18112] | 6.7 (6.4)  5^c,d^ [0-35] | 89^c,d^ (93.7) | 907.6 (1021.3)  509.3^c^ [0-3247.7] | 4.6 (4.1)  4^c,d^ [0-13] | 13 (76.5) |
| Dural Nodule | Gd+ Delayed 3T FLAIR | 15.5 (30.2)  0^c^ [0-109] | 0.6 (0.9)  0^b,c^ [0-3] | 20^b,c^ (33.3) | 9.5 (17.9)  0 [0-67] | 0.4 (0.6)  0 [0-2] | 6 (37.5) |
|  | Gd+ Early 7T FLAIR | 32.2 (75.1)  6.2 [0-628.7] | 1.1 (1.4)  1^b,d^ [0-9] | 55^b^ (57.9) | 25.9 (56.6)  0 [0-226.5] | 0.9 (1.3)  0 [0-4] | 7 (41.2) |
|  | Gd+ Delayed 7T FLAIR | 28.6 (37.8)  12.6^c^ [0-207.9] | 1.9 (2.2)  1^c,d^ [0-8] | 67^c^ (70.5) | 16.6 (27.8)  1.7 [0-96.4] | 1 (1.2)  1 [0-4] | 9 (52.9) |
| Total | Gd+ Delayed 3T FLAIR | 445.1 (985.7)  109.7^b,c^ [0-109.7] | 3.2 (3.9)  2^b,c^ [0-19] | 42^b,c^ (70) | 358.1 (751.8)  22.9 [0-2889] | 2.8 (3.6)  1^c^ [0-12] | 11 (68.8) |
|  | Gd+ Early 7T FLAIR | 800.7 (1379.8)  249.3^b,d^ [0-9919.9] | 5.4 (4.6)  4^a,b,d^ [0-22] | 85^b,d^ (89.5) | 198.2 (252.2)  115.3 [0-866.4] | 2.7 (2.7)  1**^a,d^** [0-8] | 12 (70.6) |
|  | Gd+ Delayed 7T FLAIR | 1287.6 (2086.9)  646.7^c,d^ [0-18134.2] | 10.1 (6.9)  8^c,d^ [0-36] | 94^a,c,d^ (98.9) | 968.1 (1064.7)  605.7^c,d^ [0-3447.1] | 6.6 (4.9)  7^c,d^ [0-18] | 15^a^ (88.2) |

HC = healthy control, MS = multiple sclerosis, ME = meningeal enhancement

Values shown are mean (SD) and median [minimum-maximum]. Mean shown for informational purposes, but statistical tests performed using non-parametric testing, and thus statistical significance to be shown for median values.

“**^a^**” = p < 0.05 for comparison of HC and MS, “^b^” = p < 0.05 for comparison between Gd+ Delayed 3T FLAIR and Gd+ Early 7T FLAIR, “^c^” = p < 0.05 for comparison between Gd+ Delayed 3T FLAIR and Gd+ Delayed 7T FLAIR, “^d^” = p < 0.05 for comparison of Gd+ Early 7T FLAIR and Gd+ Delayed 7T FLAIR.
