# Supplementary material for: Meningeal contrast enhancement in multiple sclerosis: Assessment of field strength, acquisition delay, and clinical relevance
Source: PLoS One. 2024 May 29;19(5):e0300298. doi: 10.1371/journal.pone.0300298 (PMC11135724; doi:10.1371/journal.pone.0300298)
Supplement: S2 Table — (DOCX) [file pone.0300298.s003.docx]

**S2 Table: Demographic and clinical characteristics by presence of LMPE in the MS cohort.**

|  | | LMPE | | | | | |
| --- | --- | --- | --- | --- | --- | --- | --- |
|  | | Gd+ 3T  LMPE +  N = 38 | Gd+ 3T LMPE -  N = 22 | Gd+ Early 7T  LMPE +  N = 79 | Gd+ Early 7T LMPE -  N = 16 | Gd+ Delayed 7T  LMPE +  N = 94 | Gd+ Delayed 7T LMPE -  N = 1 |
| Mean Age (SD) | | 45.4 (10.4) | 44.5 (9.9) | 46.4* (10.5) | 39.6* (9.0) | 45.2 (10.6) | 53 (n/a) |
| Median Disease Duration [min-max] | | 14.0 [1.8-33.3] | 12.5 [0.4-24.2] | 11.2 [1.5-47.8] | 12.4 [0.44, 23.4] | 11.3 [0.4-47.8] | 17.9 [n/a] |
| Female Gender (%) | | 30* (79.0) | 11* (50) | 54 (68.4) | 12 (75) | 65 (69.1) | 1 (n/a) |
| Disability Scales | Median EDSS  [min-max] | 2.5 [0-6.5] | 2.25 [0-6.5] | 2.5 [0-6.5] | 2.5 [0-6.5] | 2.5 [0-6.5] | 4.0 [n/a] |
|  | Median 9HPT  [min-max] | 21.2 [15.6-425.7] | 22.4 [17.6-231.1] | 22.6 [15.6-425.7] | 22.6 [17.6-65.0] | 22.6 [15.6-425.7] | 20.5 [n/a] |
|  | Median T25W [mix-max] | 4.9 [3.7-161] | 5.0 [3.7-161] | 5.4 [3.2-161] | 5.1 [3.4-24.1] | 5.3 [3.2-161] | 6.4 [n/a] |
|  | Mean SDMT (SD) | 55.0 (16.0) | 52.2 (12.5) | 53.31 (15.05) | 57.55 (15.27) | 54.0 (15.1) | 49 (n/a) |
|  | Median PASAT [min-max] | 47 [0-59] | 50 [26-58] | 45 [0, 60] | 42.5 [23, 55] | 45 [0-60] | 49 [n/a] |

LMPE = leptomeningeal and paravascular enhancement, SD = standard deviation. EDSS = Expanded Disability Status Scale, 9HPT = Nine Hole Peg Test (in seconds), T25W = Timed 25-foot walk (in seconds), SDMT = Symbol Digit Modalities Test (# correct).

Wilcoxon rank sum test was used for all values shown as median, t-test for those shown as mean, and proportions were tested by Chi-square or Fisher’s exact test. * = p < 0.05. Note: statistical tests not completed for Gd+ Delayed 7T, as only 1 subject in LMPE- group. SD and range shown as n/a for the same reason.
