# Supplementary material for: Meningeal contrast enhancement in multiple sclerosis: Assessment of field strength, acquisition delay, and clinical relevance
Source: PLoS One. 2024 May 29;19(5):e0300298. doi: 10.1371/journal.pone.0300298 (PMC11135724; doi:10.1371/journal.pone.0300298)
Supplement: S3 Table — (DOCX) [file pone.0300298.s004.docx]

**Table S3: Comparison of rates of MCE by DMT class**

| MCE Type | Low/Moderate Efficacy DMT | High Efficacy DMT |
| --- | --- | --- |
| Gd+ Delayed 3T FLAIR LME | 7 (25.0%) | 5 (18.5%) |
| Gd+ Delayed 3T FLAIR LMPE | 17 (60.7%) | 17 (63.0%) |
| Gd+ Delayed 3T FLAIR PDE | 16 (57.1%) | 19 (70.4%) |
| Gd+ Early 7T FLAIR LME | 17 (50.0%) | 17 (40.5%) |
| Gd+ Early 7T FLAIR LMPE | 29 (85.3%) | 35 (83.3%) |
| Gd+ Early 7T FLAIR PDE | 30 (88.2%) | 36 (85.7%) |
| Gd+ Delayed 7T FLAIR LME | 18 (52.9%) | 21 (50.0%) |
| Gd+ Delayed 7T FLAIR LMPE | 34 (100%) | 41 (97.6%) |
| Gd+ Delayed 7T FLAIR PDE | 34 (100%) | 40 (95.2%) |

DMT = disease modifying therapy

Proportion comparisons performed by Chi-square testing. * = p < 0.05. Note: no significant differences found.
