# Supplementary material for: Meningeal contrast enhancement in multiple sclerosis: Assessment of field strength, acquisition delay, and clinical relevance
Source: PLoS One. 2024 May 29;19(5):e0300298. doi: 10.1371/journal.pone.0300298 (PMC11135724; doi:10.1371/journal.pone.0300298)
Supplement: Attachment — Submitted filename: Titelbaum comments and suggestions for Harrison et al.docx [file pone.0300298.s005.docx]

Specific comments and suggestions:

This study brings another step forward our understanding of MCE in MS. The novel approach looks at MCE in leptomeningeal (LME) and non leptomeningeal compartments (PDE, LMPE) thus further refining precision of definition of leptomeningeal and non-leptomeningeal enhancement, and in that endeavor the topic is worthy of publication. Their imaging analysis in terms of MRI method is thorough and complete. However there are several areas whereby re-categorization of meningeal compartments would have been preferable.

Specifically, separate LME, PE (paravascular), DE (dural) subcompartments, and then looking at combining subcompartments PDE=PE +DE and LMPE=LME+PE) would have been preferred. PE and DE are observable at MRI but were not assessed at the original pathological analysis by Magliozzi and Howell, and so the current manuscript offers further insight.

1. BACKGROUND/PURPOSE:

The authors do a thorough comparison of MCE (LME/PDE) imaging detection between 3T and 7T, and compare early vs delayed acquisition timing at 7T only.

Two time points at 7T and a single delayed-only time point at 3T cannot be characterized as thorough investigation of acquisition timing. It is recommended that authors clarify this point in the Abstract and perhaps the Discussion.

2. INTRODUCTION

P3 line 8 authors should add “3D”: …that higher resolution 3D-FLAIR MRI…

(because 3D is an important method of CSF suppression facilitating LME visualization compared to 2D FLAIR).

3. METHODS:

The authors observed rates of MCE at early (approximately 1-2 minutes post-injection) on 7T only, and at delayed (21-23 minutes post injection) on 3T and 7T, subdivided by LME (leptomeningeal), PDE (paravascular and dural), and LMPE (LME + paravascular) compartments. Factors such as age, disease duration and disability were also reviewed.

The authors may wish to state why 21-23 minutes was chosen as delayed time.

It is also not clear not clear why dural-only enhancement and paravascular-only enhancement were not evaluated as individual subcompartments.

4. RESULTS:

P11 typo 2^nd^ line “visit” should be “visible”

Of interest is their 4 patients treated with corticosteroids. Did they show LME and PDE? Were they followed later and did follow ups show changes in LME and PDE?

5. DISCUSSION

First line “This study provides the largest set of data directly evaluating the impact of acquisition timing and field strength on MCE in MS to date.”

In fact this study measures the impact of acquisition timing only at 7T (not 3T) and comparison of field strength at 3T and 7T on delayed acquisition. The authors address this later in the Discussion but Abstract and initial Discussion should not mislead the reader that 21-23 minutes is necessarily the ideal acquisition time but rather that a delay is more optimal compared to early acquisition at 2 minutes.

6. P19 end of first paragraph:

The authors seem to be conflating T1 vs 3D-FLAIR vs high resolution as a reason for improved detection. 3D-FLAIR can detect low concentration of GD vs T1 (consider adding Mathews at al as a reference); wheras higher SNR permits improved spatial resolution at 7T further increasing detection ability. Stating the unique separate contributions of FLAIR and SNR for their contributions is preferred.

Mathews VP, Caldemeyer KS, Lowe MJ, Greenspan SL, Weber DM, Ulmer JL. Brain: gadolinium enhanced fast fluid-attenuated inversion-recovery MR imaging. Radiology 1999;211:257-63

7. The finding of higher LME rate for early 7T (47%) compared to delayed 3T (23%) is a bit surprising from the perspective of expected influence of timing of acquisition. While it is possible that the higher field strength is the reason, vendor-specific differences in FLAIR sequences introduces a large potential source of variability (as reported by Titelbaum et al) and should be discussed.

8. The authors may also wish to discuss reasons for difference from the 90% LME rate at 7T they reported in 2017 (Harrison et al)

CONCLUSION:

The authors appropriately conclude that LME is better detected on delayed 7T, confirming the results of prior studies. The authors also appropriately question LME as a relevant biomarker.

9. One of the major strengths of their work is the assessment of MCE in leptomeningeal and non-leptomeningeal compartments. There should be a statement in the conclusion acknowledging this contribution and its potential impact.
