# Supplementary material for: Meningeal contrast enhancement in multiple sclerosis: Assessment of field strength, acquisition delay, and clinical relevance
Source: PLoS One. 2024 May 29;19(5):e0300298. doi: 10.1371/journal.pone.0300298 (PMC11135724; doi:10.1371/journal.pone.0300298)
Supplement: Attachment — Submitted filename: Titelbaum commentary of Harrison et al with refs.docx [file pone.0300298.s006.docx]

COMMENTARY

Since leptomeningeal enhancement (LME) in (multiple sclerosis) MS was first described on post-gadolinium 3D-FLAIR MRI by Absinta et al in 2015 (1), the clinical implication and potential role as a diagnostic and therapeutic biomarker have been the subject of numerous investigations, often with discordant results.

The anatomic basis of a potential biomarker of meningeal inflammation was supported by earlier studies by Magliozzi et al (2007) (2) and Howell et al (2011) (3) whereby post-mortem analysis in MS patients showed the presence of B-cell predominant meningeal lymphoid follicles. Note that the anatomic preparations were stripped of dura so that only leptomeningeal lymphoid aggregates were analyzed. Magliozzi (2010) further defined a gradient of cortical inflammation in MS thought related to the proximity to meningeal lymphoid follicles (4). Adding to the initial excitement for a role of LME, two of the cases described by Absinta went to post-mortem assessment showing areas of LME in proximity to meningeal follicles (1).

The physiologic basis of LME detection on MRI is based on the selective visual identification on 3D-FLAIR imaging of low concentrations of gadolinium from adjacent vascular leakage, presumably inflammatory-mediated, and temporarily retained in localized pools. Low-concentrations of gadolinium are visible on 3D-FLAIR but typically not seen on post-contrast T1 imaging which are instead more sensitive to higher concentrations of gadolinium (5).

Some studies have reported higher disability scores and cortical atophy in MS patients with LME, though association of LME with cortical demyelinating lesions have been inconsistent. LME has also been reported in other inflammatory diseases and healthy controls (HCs) (6). Indeed, LME has been reported in 28% of older patients without known inflammatory illness, suggesting that aging may be a significant contributor to their occurrence (7).

The subsequent assessment of LME by multiple investigators has been confounded by numerous factors including occurrence in HCs, potential differences between relapsing and progressive MS subpopulations, variability of imaging protocols including magnet field strength, MRI scanner vendor-specific FLAIR sequence variability, timing of post-injection image acquisition, gadolinium subtype and dosage, clinical administration of corticosteroids, and the confounding of LME versus dural and perivascular enhancement.

The identification of an inflammatory imaging biomarker is a ‘holy grail’ if indeed a true biomarker can be found. While several cases of LME attenuation by intravenous corticosteroids have been reported, likely transient (1, 8), there has been failure of most currently available DMTs to modulate LME. Indeed, even intrathecal CD-20 inhibitors, the most likely candidates to control B-cell inflammation, have failed to alter LME in a small cohort (9). A recent study reported LME resolution in a small percent of MS patients treated with anti-CD-20 (10), but results may in part be confounded by steroid administration, so that confirmation of this observation is warranted. BTK inhibitors have been shown to reduce LME in mice (11) and results in human subjects are anxiously awaited. Until a pharmacologic agent is found to attenuate LME, the utility as a biomarker becomes limited. Finally, the occurrence in HCs calls into question whether LME is truly a biomarker at all versus an epiphenomena. Indeed, it has been suggested that LME may merely represent pooling of contrast into localized lymphatic pockets rather than the result of inflammation (12).

The current work by Harrison et al (13) further advances our understanding of meningeal contrast enhancement by measuring the presence of LME and dural enhancement comparing 3T vs 7T MR field strengths, in both MS and healthy controls. Their imaging methodology is thorough, comparing T1, pre- and post-Gd 3D-FLAIR sequences, subtraction, and segmentation post-processing analysis. Compared to other prior research, their work is novel in that they measure enhancement in leptomeningeal (LME) and non-leptomeningeal subcompartments including paravascular and dural locations in MS patients. Potential confounding variables include different vendors for 3T vs 7T (Siemens vs Phillips, respectively), and selection of 21-23 minutes post-injection delay. Indeed, prior investigators have found substantial variability of visibility of LME by different vendor FLAIR sequences (8), thus potentially limiting conclusions in this study of different sensitivity of 3T vs 7T. Indeed, vendor FLAIR differences could explain why the authors found higher rates of LME at early 7T acquisition (47%) compared to 3T delayed acquisition (23%). Reported heterogeneity in peak time to enhancement following injection is a possible source of error, though likely minor. Differences in gadolinium preparation (Gadoteridol used for 7T vs gadobutrol used for 3T) likely played an insignificant source of variability.

The authors rightly distinguish between leptomeningeal enhancement (LME) and paravascular + dural enhancement (PDE), but also add a category “LMPE” combining both LME and paravascular enhancement (PE) to account for vascular traversing of the subarachnoid space. It is not clear why the authors did not also measure dural-only enhancement. That LME and PDE in MS vs HC did not reach clinical significance, whereas LMPE (LME + PE) did reach significance, may have been statistically related to sample size thresholds created by the addition of two groups, unmasking leptomeningeal contribution from within the paravascular space. Furthermore, the histologic and pathologic substrate of PDE likely is related to glymphatic drainage rather than leptomeningeal leakage (12), though comparison between MS and HC contributes to our knowledge base.

In their discussion the authors rightly point out that the association of LME with inflammation is not clear, and also conclude on the basis of their data, as well as others showing correlation of LME and EDSS, that LME may be more associated with aging rather than inflammation. The authors also identify that patients with LMPE were older than those without LMPE.

Another compartment of leakage-related enhancement that has been observed on Gd-3D-FLAIR MRI but insufficiently studied is choroidal fissural enhancement (8). Magliozzzi noted increased concentrations of lymphoid follicles along the choroidal fissures in MS (2), and recent studies have observed increased choroid plexus volume correlated to increased inflammatory state (14). These observations raise the question of whether choroidal fissural leakage seen on MRI could be an independent biomarker of inflammation? It would thus be interesting to know if choroidal fissural enhancement was present in Harrison’s cohorts, and indeed if threshold concentrations could be quantified by their post-processing segmentation analysis. The authors’ cohorts of MS patients and HCs who each received gadolinium injections 1 week apart may contain valuable data they would allow assessment of this question.

The compartmentalized approach to measure MCE is a step forward in our understanding of meningeal physiology and inflammation. Future assessment of LME, PE, DE, and choroidal plexus leakage may serve to clarify true correlates of inflammatory disease process versus an epiphenomena.

References:

1.Absinta M, Vuolo L, Rao A, Nair G, Sati P, Cortese IC, et al. Gadolinium-based MRI characterization of leptomeningeal inflammation in multiple sclerosis. Neurology. 2015;85(1):18-28.

2. Magliozzi R, Howell O, Vora A, Serafini B, Nicholas R, Puopolo M, et al. Meningeal B-cell follicles in secondary progressive multiple sclerosis associate with early onset of disease and severe cortical pathology. Brain. 2007;130(Pt4):1089-104.

3. Howell OW, Reeves CA, Nicholas R, Carassiti D, Radotra B, Gentleman SM, et al. Meningeal inflammation is widespread and linked to cortical pathology in multiple sclerosis. Brain. 2011;134(Pt 9):2755-71.

4. Magliozzi R, Howell OW, Reeves C, Roncaroli F, Nicholas R, Serafini B, et al. A Gradient of neuronal loss and meningeal inflammation in multiple sclerosis. Ann Neurol. 2010;68(4):477-93.

5. Mathews VP, Caldemeyer KS, Lowe MJ, Greenspan SL, Weber DM, Ulmer JL. Brain: gadolinium enhanced fast fluid-attenuated inversion-recovery MR imaging. Radiology 1999;211:257-63.

6. Absinta M, Cortese ICM, Vuolo L, Nair G, de Alwis MP, Ohayon J,et al. Leptomeningeal gadolinium enhancement across the spectrum of neuroinflammatory diseases. Neurology 2017;88:1-6.

7. Freeze WM, van der Thiel M, de Bresser J, Klijn CJM, van Etten ES, Jansen JFA, et al. CSF enhancement on postcontrast fluid-attenuated inversion recovery images; a systematic review. Neuroimage Clin. 2020;28:102456.

8. Titelbaum DS, Engisch R, Schwartz ED, Napoli SQ, Sloane JA, Samaan S, et al. Leptomeningeal Enhancement on 3D-FLAIR MRI in Multiple Sclerosis: Systematic Observations in Clinical Practice. J Neuroimaging. 2020;30(6):917-29.

9. Bhargava P, Wicken C, Smith MD, Strowd RE, Cortese I, Reich DS, et al. Trial of intrathecal rituximab in progressive multiple sclerosis patients with evidence of leptomeningeal contrast enhancement. Mult Scler Relat Disord. 2019;30:136-40.

10. Friedli C, Wagner F, Hammer HN, Kamber N, Wiest R, Diem L, et al. Leptomeningeal enhancement under different MS immunotherapies: A monocentric retrospective cohort study of 214 patients. Mult Scler. 2023;29(1):63-73.

11. Bhargava P, Kim S, Reyes AA, Grenningloh R, Boschert U, Absinta M, et al. Imaging meningeal inflammation in CNS autoimmunity identifies a therapeutic role for BTK inhibition. Brain. 2021;144(5):1396-408.

12. Wu CH, Lirng JF, Ling YH, Wang YF, Wu HM, Fuh JL, et al. Noninvasive Characterization of Human Glymphatics and Meningeal Lymphatics in an in vivo Model of Blood-Brain Barrier Leakage. Ann Neurol. 2021;89(1):111-24.

13. Harrison DM, Allette YM, Zeng Y ,Cohen A, Dahal S, Choi S, et al. Meningeal contrast enhancement in multiple sclerosis: assessment of field strength, acquisition delay, and clinical relevance. --------------202X

14. Ricigliano VAG, Morena E, Colombi A, Tonietto M, Hamzaoui M, Poirion E, et al. Choroid plexus enlargement in inflammatory multiple sclerosis: 3.0T MRI and translocatorprotein PET evaluation. Radiology 2021;301(1):166-77.
