# Supplementary material for: Meningeal contrast enhancement in multiple sclerosis: Assessment of field strength, acquisition delay, and clinical relevance
Source: PLoS One. 2024 May 29;19(5):e0300298. doi: 10.1371/journal.pone.0300298 (PMC11135724; doi:10.1371/journal.pone.0300298)
Supplement: Attachment — Submitted filename: PLOS One 7T 3T MCE Paper - Response to Reviewers.docx [file pone.0300298.s007.docx]

Dear Reviewers,

Thank you for taking the time to review our paper, “Meningeal contrast enhancement in multiple sclerosis: assessment of field strength, acquisition delay, and clinical relevance”. We appreciate the thoughtful and thorough evaluation of this paper. We have made the suggested modifications, which can be found in track changes format. To aid evaluation of the revision, we have also responded directly to your comments/suggestions below. Our responses can be found in *italic* font.

Regards,

Daniel M. Harrison on behalf of the authors of “Meningeal contrast enhancement in multiple sclerosis: assessment of field strength, acquisition delay, and clinical relevance.”

Reviewer #1:

Specific comments and suggestions:

This study brings another step forward our understanding of MCE in MS. The novel approach looks at MCE in leptomeningeal (LME) and non leptomeningeal compartments (PDE, LMPE) thus further refining precision of definition of leptomeningeal and non-leptomeningeal enhancement, and in that endeavor the topic is worthy of publication. Their imaging analysis in terms of MRI method is thorough and complete. However there are several areas whereby re-categorization of meningeal compartments would have been preferable.

Specifically, separate LME, PE (paravascular), DE (dural) subcompartments, and then looking at combining subcompartments PDE=PE +DE and LMPE=LME+PE) would have been preferred. PE and DE are observable at MRI but were not assessed at the original pathological analysis by Magliozzi and Howell, and so the current manuscript offers further insight.

*We thank the reviewers for their support of our approach. The suggestion to perform analyses for paravsacular and dural foci separately is similar to our own thought process, and, in fact, this is what was done. Data for group and protocol comparisons for nodular, spread/fill, paravascular, and dural foci individually is found in S1 Table (previously Supplemental Table S1 in the original appendix file). We have added some additional language to the results sections for protocol comparisons and group comparisons to make this more clear/explicit. References to results for individual foci types (paravacular, for example) were already in the original section on correlations with disability.*

1. BACKGROUND/PURPOSE:

The authors do a thorough comparison of MCE (LME/PDE) imaging detection between 3T and 7T, and compare early vs delayed acquisition timing at 7T only.

Two time points at 7T and a single delayed-only time point at 3T cannot be characterized as thorough investigation of acquisition timing. It is recommended that authors clarify this point in the Abstract and perhaps the Discussion.

*This is an excellent point, which does limit the widespread applicability of the acquisition timing conclusion. Unfortunately, as this study was conducted as part of a larger, multi-objective study, with many sequences needed for various goals, we were limited in what sequences we could include after contrast on the 3T protocol, and had to make the hard decision not to acquire an early acquisition 3T FLAIR as to make room for other sequences. In accordance with your suggestion, we have removed the word “comprehensive” from the abstract and introduction, and we have also added some additional comments in the later part of the discussion to acknowledge this limitation.*

2. INTRODUCTION

P3 line 8 authors should add “3D”: …that higher resolution 3D-FLAIR MRI…

(because 3D is an important method of CSF suppression facilitating LME visualization compared to 2D FLAIR).

*This has been added.*

3. METHODS:

The authors observed rates of MCE at early (approximately 1-2 minutes post-injection) on 7T only, and at delayed (21-23 minutes post injection) on 3T and 7T, subdivided by LME (leptomeningeal), PDE (paravascular and dural), and LMPE (LME + paravascular) compartments. Factors such as age, disease duration and disability were also reviewed.

The authors may wish to state why 21-23 minutes was chosen as delayed time.

It is also not clear not clear why dural-only enhancement and paravascular-only enhancement were not evaluated as individual subcompartments.

*We have added language to the methods section to explain the timing of the contrast delay, which was both due to prior literature on enhancement kinetics and practical needs based on the length of sequences being acquired before the Delayed 7T and Delayed 3T FLAIR images. References to the kinetics component of this was already in the original discussion section, page 23.*

*As stated above, dural-only and parvascular-only analyses were completed and are found in Supplemental tables.*

4. RESULTS:

P11 typo 2^nd^ line “visit” should be “visible”

Of interest is their 4 patients treated with corticosteroids. Did they show LME and PDE? Were they followed later and did follow ups show changes in LME and PDE?

*The typo has been corrected.*

*In response to this query regarding corticosteroids and Reviewer #2’s suggestion regarding the effect of DMTs, we have added a new section to the results, some language in the discussion, and a new S3 Table, all of which address the effects of treatment.*

5. DISCUSSION

First line “This study provides the largest set of data directly evaluating the impact of acquisition timing and field strength on MCE in MS to date.”

In fact this study measures the impact of acquisition timing only at 7T (not 3T) and comparison of field strength at 3T and 7T on delayed acquisition. The authors address this later in the Discussion but Abstract and initial Discussion should not mislead the reader that 21-23 minutes is necessarily the ideal acquisition time but rather that a delay is more optimal compared to early acquisition at 2 minutes.

*The language implying that 23 minutes is ideal has been removed from the early part of the discussion.*

6. P19 end of first paragraph:

The authors seem to be conflating T1 vs 3D-FLAIR vs high resolution as a reason for improved detection. 3D-FLAIR can detect low concentration of GD vs T1 (consider adding Mathews at al as a reference); wheras higher SNR permits improved spatial resolution at 7T further increasing detection ability. Stating the unique separate contributions of FLAIR and SNR for their contributions is preferred.

Mathews VP, Caldemeyer KS, Lowe MJ, Greenspan SL, Weber DM, Ulmer JL. Brain: gadolinium enhanced fast fluid-attenuated inversion-recovery MR imaging. Radiology 1999;211:257-63

*We apologize if this paragraph was worded in a confusing manner. The intent of the paragraph was not to discuss comparisons of T1 vs FLAIR for detection of low concentrations of Gd. This topic has been well proven elsewhere (including the reference above). The intent was to state that when comparing various 3D-FLAIR methods, improvements in resolution and SNR via 7T vs 3T should be advantageous. We have added the words 3D-FLAIR in appropriate spots to ensure that this is more clear.*

7. The finding of higher LME rate for early 7T (47%) compared to delayed 3T (23%) is a bit surprising from the perspective of expected influence of timing of acquisition. While it is possible that the higher field strength is the reason, vendor-specific differences in FLAIR sequences introduces a large potential source of variability (as reported by Titelbaum et al) and should be discussed.

*This has been added as a limitation to this study in the discussion.*

8. The authors may also wish to discuss reasons for difference from the 90% LME rate at 7T they reported in 2017 (Harrison et al)

*Thank you for the observation. Our group has had multiple publications subsequent to that original description of LME at 7T (refs: Ighani et al, Mizell et al, Jonas et al, Dahal et al in our reference list), all of which were larger in sample size and size/breadth, and had LME rates similar to the current manuscript. The original description was in a very small cohort and appears to have been an outlier due to sample bias. Given the overwhelming number of publications from our group and others at 7T that have similar data, we feel it would not be worth time/space in the current manuscript to single this one paper out.*

CONCLUSION:

The authors appropriately conclude that LME is better detected on delayed 7T, confirming the results of prior studies. The authors also appropriately question LME as a relevant biomarker.

9. One of the major strengths of their work is the assessment of MCE in leptomeningeal and non-leptomeningeal compartments. There should be a statement in the conclusion acknowledging this contribution and its potential impact.

*We thank you for this endorsement. Non-leptomeningeal compartments have been analyzed in MS studies in two of our prior publications on this topic (Jonas et al, Dahal et al in the reference list) and by Hildesheim et al (Mult Scler Relat Disord. 2021 Jan;47:102653.). Thus, we feel it would be inappropriate to suggest in this paper that such an analysis is a novel contribution to the literature.*

Reviewer #2:

This paper describes a cross-sectional analysis of leptomeningeal and dural contrast enhancement detected on 3T and 7T MRI compared to healthy controls. The study included 95 MS subjects and 17 controls. The study assessed the detection properties regarding the time of contrast administration. It is found that LME is more common in patients with MS and better detected in 7T scanners with postponed image acquisition.
The study is of high scientific value, because not so many research groups work on methodology of LME detection and scans' acquisition. Specifically, the use of 7 Tesla makes the study of great value due to high resolution of the scan and detection of different types of pathology. The results clearly state that postponed 7 Tesla imaging is more sensitive than the early one.

Several issues are worth to discuss:
1. A surprisingly high amount of LME detected in the control group. These findings are contradicting to another studies on 7 Tesla machine where HC group were positive in 6.7%. I would like the authors to discuss more on these results since they can lead to very forthcoming conclusions regarding the specificity of this biomarker. It is clearly stated, though, that LME may be found in another diseases, e.g. non-inflammatory CNS disorders, and even in healthy controls, so such a high percentage of LME and PDE in the control group would refer to eligibility criteria for controls and to review of their medical history.

*We have added some additional language to the paragraph discussing the HC findings to discuss differences between this and other studies. We also added a line about exclusion criteria to the methods section.*

2. Several factors may have had the impact of LME prevalence in this study and low difference to the HC group. For example, the a great amount of patients with MS in the study received anti-B-cell therapy and anti-VLA-4 therapy, that may decrease the prevalence of LME (although studies on that are still running). It is worth considering a therapy type or line (1st line and 2nd line) to include in regression models to account for.

Also, this patient group was rather moderately disabled, since the median EDSS score was 2.5. Some studies showed that patients of early stages can show no LME while it's accumulation appears on later stages. It would be interesting to see the numbers of LME in patients with different EDSS scores as a part of this research process. This may also be an interesting topic for further research.

*Thank you for the suggestions. Given the suggestion to evaluate treatment more specifically, we have added an analysis of treatment section to the results, discussion, and S3 Table.*

*Evaluation of the relationship between EDSS scores and meningeal enhancement were already performed in our analysis (see Table 3 and corresponding results section). We found no relationship between EDSS scores and meningeal enhancement. Given our findings regarding age, we suggest that the previous reports of associations may have been age-mediated.*

3. Only few MRI Departments have access to 7 Tesla MRI, so, if this biomarker comes to be used routinely, a 3 Tesla will be the main machine used for it. I would like the authors to describe why they didn't use early time 3 Tesla sequence and only used delayed 3 Tesla acquisition. This may be of additional knowledge on the methodology.

It would be also of some importance if the authors would describe why specifically 21 and 23 minutes were selected as timepoints for MRI scans. Other labs use other timeframes and it would great if authors would describe their decision-making process.

Finally, I can conclude that this papers is of high scientific and practical value and requires only minor changes to make several issues more clear.

*Thank you for the comments and suggestions. In light of both these comments and similar suggestions from Reviewer 1, we have added more information on the choice of timing and lack of early acquisition on the 3T scanner. Please see our responses to Reviewer 1 for more details.*
